# Isolated diplopia associated with calcineurin inhibitor therapy in a patient with idiopathic membranous nephropathy: a case report

**DOI:** 10.1186/s12882-016-0309-4

**Published:** 2016-08-19

**Authors:** Ashwani K. Gupta, Nader Bahri

**Affiliations:** Department of Nephrology, University of Florida, 655 W 8th St, Jacksonville, FL 32209 USA

**Keywords:** Calcineurin inhibitors, Neurotoxicity, Diplopia

## Abstract

**Background:**

Neurotoxicity is a common side effect of treatment with calcineurin inhibitors. Tremors are frequently reported as the most common manifestation. Variable presentations can include headaches, seizures, visual hallucinations or blindness. Sixth nerve palsy has been reported in previous cases of bone marrow and cardiac transplant patients receiving calcineurin inhibitors. In many of these previously reported cases, the drug was administered intravenously and very high drug levels were found.

**Case presentation:**

We report the first case of isolated diplopia in a patient being treated for idiopathic membranous glomerulonephritis. This is also the first report where the neurotoxicity induced by initial tacrolimus therapy persisted with subsequent cyclosporine therapy, two structurally different calcineurin inhibitors which share a common mechanism of action. In our case toxicity occurred after 3 months of therapy despite low serum concentrations and the symptoms resolved completely after discontinuation of the drugs.

**Conclusion:**

Our case provides further evidence that the neurotoxicity is a result of calcineurin inhibition. Monitoring of serum concentrations of these drugs has not been correlated with toxicity. The mean duration to onset of symptoms can be as much as 70 days suggesting accumulation of the drug in the central nervous system plays a role. Recognition of this condition is important for prompt diagnosis and appropriate management.

## Background

Calcineurin inhibitors cyclosporine (CyA) and tacrolimus (FK506) are widely used immunosuppressive drugs used to treat transplant recipients, autoimmune diseases and nephrotic syndrome. CyA and FK506 bind to cyclophilin and FK binding protein respectively and the resulting complex inhibits calcineurin. Besides lymphocytes, calcineurin is also found in abundance in the nervous tissues. These drugs are lipophilic and move across the blood brain barrier easily. Diverse neurotoxicities ranging from tremors (most common, up to 40 %), headache, altered mental status, hallucinations, psychosis, peripheral neuropathy, seizures, cerebellar ataxia and leukoencephalopathy have been reported in the literature. In most cases the calcineurin therapy induced neurotoxicity is reversible after withdrawal of the medication. We report for the first time a case of isolated diplopia in a patient with idiopathic membranous nephropathy on treatment with calcineurin inhibitors.

## Case presentation 

**Fig. 1:**
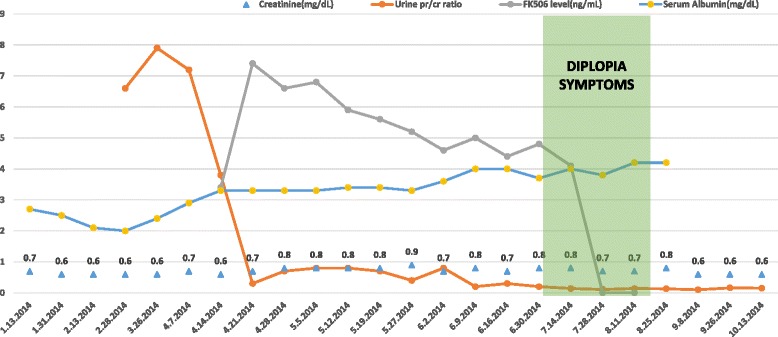
Patient's clinical course

Our case is a previously healthy 42 year-old Caucasian female with biopsy proven idiopathic membranous nephropathy (MGN) who was being treated with FK506 and prednisone for nephrotic syndrome. She was a never smoker and did not have a prior history of hypertension or diabetes. She tested negative for ANA, ANCA vasculitis, HIV, hepatitis B and C. Her kidney biopsy showed typical features of membranous glomerulonephritis in addition to positive staining with the anti-phospholiapse A2 receptor antibody which identifies idiopathic MGN with 97 % specificity [1]. Serum Tacrolimus levels were maintained between 6 and 8 ng/mL. She responded favorably to treatment and her initial urine protein/creatinine ratio of 8 gm/gm declined to less than 1 gm/gm within the initial 2 weeks of therapy. Her serum albumin level of 2 gm/dL prior to initiation of therapy improved to 3.3 gm/dL after 3 weeks of initiation of treatment (Fig. 1). The patient also developed hypertension as a side effect of calcineurin inhibitor therapy and was treated with Losartan 100 mg PO daily. Her blood pressure remained well controlled throughout the treatment period and hypertension resolved once therapy was discontinued. After 3 months of therapy she presented with diplopia. She was in complete remission at this time and her serum albumin had normalized to 4 gm/dL. Her clinical course is depicted in Fig. 1. The diplopia was gradual in onset, binocular and vertical and more prominent in the later part of the day. The patient was seen for an ophthalmologic evaluation. Her visual acuity was 20/20. Pupils were equal and reactive to light and accommodation. No nystagmus or ptosis was observed. Visual fields and color vision was also normal in both eyes. Assay for acetylcholine receptor antibody done to rule out myasthenia gravis was negative. A CT scan of the brain done to rule out an infarct was normal. The diplopia persisted for over 4 weeks while the patient remained compliant with her medications despite the side effects. The symptoms persisted even when her tacrolimus dose was reduced and repeat levels were between 4 and 5 ng/mL. 3 weeks after onset of diplopia she was switched to low dose CyA in anticipation that similar side effects may not be observed. Trough CyA levels were 44 ng/mL and 59 ng/mL on two occasions but her symptoms did not resolve. A consultation with neuro-ophthalmology was sought and the patient was instructed to discontinue CyA. The symptoms completely resolved 6 days after stopping CyA. Her nephrotic syndrome remains in remission till date.

## Conclusion

A review of the literature found 6 cases of eye movement disorders in association with calcineurin inhibitor therapy. The relevant clinical findings of these are summarized in Table 1. All of these occurred in patients with solid organ [2] or bone marrow transplants [3, 4] and were associated with high levels of CNIs. Our case is unusual in several respects. This is the first reported case in a patient with nephrotic syndrome. Also, this is the first reported case where manifestations persisted even when the patient was switched from FK506 to low dose CyA. CyA and FK506 are structurally different but share a common mechanism of action. Openshaw et al. [4] postulated that eye movement disorders in bone marrow transplant patients could be a result of combined treatment with CyA and ganciclovir. Our case reinforces the belief that neurotoxicity is mediated through calcineurin inhibition. In the setting of profound hypoalbuminemia like seen in our patient early in the course of disease, whole blood concentration of tacrolimus have a very variable relationship to the unbound fraction of the drug. In general, aiming for the same whole blood concentration can vastly underestimate the unbound fraction thereby leading to toxicity [5]. In our patient the diplopia developed after serum albumin had normalized. However, the preceding hypoalbuminemia may have led to greater accumulation of the drug in the central nervous system, thereby predisposing the patient to toxicity. This case highlights the variable and unpredictable nature of calcineurin inhibitor neurotoxicity. A predictable correlation between drug levels and neurotoxicity has not been found previously, but it may be related to total amount of drug and its accumulation within the central nervous system. In all previously reported cases the average time to onset was 74 days is similar to our case. A hyper intense T2 weighted MRI lesion in the occipital lobe (reversible posterior cerebral edema syndrome) is commonly reported as an indicator of neurotoxicity. Similar MRI findings can also be seen in hypertensive encephalopathy caused by vasospasm, loss of auto regulation and local edema. Diplopia can also be caused cranial nerve III, IV or VI palsy or vascular phenomenon such as vasospasm or an ischemic infarct. Our patient did not have any risk factors for microvascular disease including diabetes, hypertension or smoking. Our patient had well controlled blood pressures throughout treatment and a CT of the brain was reported normal. Therefore the patient’s symptoms cannot be attributed to a vascular phenomenon. Binocular diplopia which is only present with both eyes open excludes disorders of the ocular media such as cataracts or astigmatism. Our patient underwent a complete ophthalmologic evaluation which was normal. Further, prolonged duration of symptoms and the temporal correlation between discontinuation of the drug and resolution of symptoms strengthens our belief that this was a result of drug toxicity.

**Table 1:** Cases reported in literature presenting with eye movement disorders in the setting of calcineurin inhibitor therapy

Study Title and author	Age/Sex	Diagnosis	Drug name	Days after treatment	Route of administration	Drug level	Neurological Deficit
Openshaw, Slatkin et al. 1997 [4]		Post bone marrow transplant for Acute Lymphocytic Leukemia	Cyclosporin	72	Per Oral	475 ng/ml	Unilateral six nerve palsy
Case 1	16/Female						
Case 2	37/Female	Post bone marrow transplant for Acute Lymphocytic Leukemia	Cyclosporin	73	Per Oral	1356 ng/ml	Ptosis, bilateral sixth nerve palsy
Case 3	18/Female	Acute Leukemia Mixed lineage	Cyclosporin	47	Intravenous	700 ng/ml	Ptosis, bilateral sixth nerve palsy encephalopathy
Case 4	20/Female	Chronic Myelogenous Leukemia	Cyclosporin	80	Intravenous	Not Reported	Ptosis, bilateral sixth nerve palsy encephalopathy seizure
Lai, Kerrison et al. 2004 [2]	50/Male	Post orthotopic cardiac transplant	FK506	93	Intravenous	10–20 ng/dl	Worsening headache horizontal diplopia, Bilateral Inter nuclear opthalmoplegia
Oliverio, Restrepo et al. 2000 [3]	30/female	Post bone marrow transplant for chronic myeloid leukemia	FK506	84	Per Oral	Reported as within therapeutic range	Diplopia, horizontal nystagmus, and bilateral sixth nerve palsy. visual hallucinations

## Abbreviations

CyA, cyclosporine; FK506, tacrolimus; iMGN, idiopathic membranous nephropathy
